# Effect of post-deposition aluminizing on the corrosion and mechanical behavior of WAAM-fabricated stainless steel and Ni-based superalloy

**DOI:** 10.1038/s41598-025-30389-4

**Published:** 2025-12-06

**Authors:** Ali Günen, Uğur Gürol, Ahmet Çakan, Mustafa Koçak, Gürel Çam, Hasan Yildizhan, Ahmed Alsaadi, João Gomes

**Affiliations:** 1 Department of Metallurgy and Materials Engineering, Iskenderun Technical University, Faculty of Engineering and Natural Sciences, Iskenderun, Hatay Turkey; 2 Department of Metallurgy and Materials Engineering, Istanbul Gedik University, Faculty of Engineering, Istanbul, Turkey; 3 Istanbul Vesuvius Refrakter A.S., FOSECO Foundry Division, Kocaeli, Turkey; 4 Department of Mechanical Engineering, Mersin University, Mersin, Turkey; 5 Department of Mechanical Engineering, Istanbul Gedik University, Faculty of Engineering, Istanbul, Turkey; 6 Department of Mechanical Engineering, Iskenderun Technical University, Faculty of Engineering and Natural Sciences, Iskenderun, Hatay Turkey; 7 Energy Systems Engineering, Engineering Faculty, Adana Alparslan Türkeş Science and Technology University, Adana, 46278 Turkey; 8 University College London, London, UK; 9 Faculty of Engineering and Sustainable Development, University of Gävle, Gävle, 801 76 Sweden

**Keywords:** Stainless steel, Inconel 625, WAAM, Arc-DED, Aluminizing, Corrosion, Engineering, Materials science

## Abstract

The demand for corrosion-resistant and mechanically reliable metallic components in marine, chemical processing, and energy conversion industries has encouraged the integration of additive manufacturing into industrial production. Wire Arc Additive Manufacturing enables the fabrication of medium- to large-scale complex metallic structures at low cost; however, the high thermal input and layer-by-layer deposition commonly lead to elemental segregation, porosity, and nonuniform microstructures that degrade corrosion performance. This study investigates the influence of a post-deposition aluminizing treatment on the surface characteristics and corrosion behavior of stainless steel ER307 and nickel-based superalloy Inconel 625 produced by Wire Arc Directed Energy Deposition. Microstructural evolution, phase transformation, hardness distribution, and corrosion behavior in a 3.5% sodium chloride environment were examined through microscopy, X-ray diffraction, hardness testing, and electrochemical analysis. The aluminizing process generated localized surface porosity and limited non-uniformity aluminide coatings of approximately 40–50 μm thickness, reduced surface roughness, and markedly improved surface hardness. Electrochemical assessments demonstrated substantial enhancements in corrosion resistance, including a 2.3-fold improvement for stainless steel and a 13.9-fold improvement for Inconel 625. These findings reveal that post-deposition aluminizing effectively mitigates intrinsic surface defects and microchemical heterogeneity, enabling significantly improved durability in chloride-containing environments. This work provides a straightforward and scalable strategy for enhancing the corrosion resistance of wire-arc-manufactured metallic structures and promotes their application in aggressive service conditions.

## Introduction

Additive manufacturing (AM) has redefined modern industrial fabrication by enabling the layer-wise construction of complex components directly from digital models. Its unprecedented ability to minimize material waste, reduce production time, and fabricate intricate geometries has positioned it at the forefront of advanced manufacturing technologies^1,2^. AM encompasses a wide range of techniques based on the controlled addition, fusion, or solidification of materials through energy sources such as plasma arcs, lasers, or electron beams. Compared to traditional manufacturing approaches, AM is increasingly replacing conventional production routes, particularly in fabricating geometrically complex parts, due to advantages such as reduced material consumption, shortened processing time, and direct one-to-one translation of Computer-Aided Design (CAD) models into physical components. As reported by the end of 2020, the global AM market was valued at nearly 21 billion USD^3,4^. Beyond fusion-based additive manufacturing processes such as selective laser melting, electron beam melting, and wire arc directed-energy deposition, several non-melting approaches have gained attention in recent years. Binder jetting enables near-net-shape fabrication through powder consolidation without melting, making it suitable for low-thermal-distortion processing of metal and ceramic components. Filament-based deposition techniques, including fused filament fabrication, allow cost-effective polymer or composite part production, although typically with lower mechanical performance than fusion-based systems. More recently, cold-spray-based additive manufacturing has emerged as a promising pathway for producing oxidation-free, fully dense metallic structures at relatively low temperatures. Despite these advances, wire-arc-based deposition remains advantageous owing to its superior deposition rates, scalability, and cost efficiency when fabricating large metallic components, motivating its wider exploration in demanding industrial sectors.

Metal additive manufacturing (MAM) processes are mainly categorized into two major types: Powder Bed Fusion (PBF) and Direct Energy Deposition (DED). In PBF techniques, high-energy lasers or electron beams are employed to selectively melt metal powders. In contrast, DED techniques are subdivided into powder-fed and wire-fed systems^5–7^, in which concentrated thermal energy—generated by lasers, plasma arcs, or electron beams—is used to melt and deposit material simultaneously throughout the manufacturing process^4–7^. Wire Arc Additive Manufacturing (WAAM), also known as Wire Arc-DED (WA-DED), represents a wire-fed variant of the DED method in which arc welding is integrated with metallic wire feedstock to deposit components layer-by-layer. WAAM is distinguished by its relatively low equipment cost, compatibility with common wire diameters, high deposition rates, and suitability for large-scale fabrication. Nevertheless, the process presents several challenges, including high heat input, dimensional inaccuracies, and relatively rough surface textures. These limitations often result in inferior mechanical strength and corrosion resistance in WAAM-fabricated components compared to those produced using alternative AM techniques or conventional processing routes^8–10^.

Recent research has actively explored ways to improve the microstructure^11,12^, mechanical performance^13,14^, wear characteristics^15–17^, and oxidation resistance^18–20^ of WAAM-processed components. For instance, Inconel 625 alloys produced via WAAM were subjected to annealing treatments at 980 °C for durations of 0.5, 1, and 2 h, followed by water quenching. These treatments affected secondary phase formation, although the grain morphology remained unchanged. The result was a modest increase in ultimate tensile strength - up to 5% - without altering the yield strength compared to untreated samples^13,14^. Although Inconel 625 is typically considered a single-phase γ-Ni solid-solution strengthened alloy, several secondary phases may precipitate during prolonged exposure at intermediate temperatures. The orthorhombic δ-phase (Ni₃Nb) and the tetragonal γ″ (Ni₃Nb) phase commonly precipitate between 650 and 900 °C, promoting matrix strengthening while reducing ductility. In addition, Nb-rich Laves phases and MC-type carbides may form at interdendritic regions, particularly under non-equilibrium solidification. These secondary phases profoundly affect service performance: while γ″ and δ precipitates enhance creep strength, the brittle Laves phase can degrade hot-workability and corrosion resistance by promoting micro-galvanic heterogeneity within the matrix. Therefore, controlling the formation and distribution of such precipitates is essential in tailoring microstructural stability and mechanical behavior in additively manufactured Inconel 625.

The improvements reported in previous studies can be attributed to several underlying mechanisms. Thermochemical treatments such as aluminizing promote inward diffusion of Al and outward diffusion of Ni, leading to the formation of stable intermetallic layers (NiAl, Ni₂Al₃) that increase surface hardness and suppress localized corrosion; such coatings also reduce elemental segregation at interdendritic regions, thereby mitigating the micro-galvanic activity known to accelerate corrosion in WAAM-processed Inconel 625. Furthermore, elevated-temperature exposure promotes stress relaxation and partial homogenization of solute clusters, enhancing mechanical stability, which collectively explains the improved tribological and corrosion performance observed in aluminized WAAM components. Consistent with these findings, Safarzade et al.^21^ applied a two-step heat treatment to Arc-DED fabricated Inconel 625—consisting of a 6-hour homogenization at 1100 °C followed by water quenching, and subsequent ageing at 700 °C for 24 h—resulting in increased hardness, yield strength, and ultimate tensile strength, accompanied by reduced elongation. Similarly, Gunen et al.^16,17^ investigated boronizing treatments at 980–1000 °C for one hour on WAAM-fabricated Inconel 625 and ER307 stainless steel, reporting substantial improvements in high-temperature wear resistance due to enhancements in microstructure and hardness. In another study, Farfan-Cabrera et al.^22^ implemented thermochemical boronizing to improve the tribological properties of WAAM-repaired low-carbon steel, achieving significant increases in hardness and wear resistance while maintaining uniform surface quality. Parallel to these observations, Gurol et al.^19^ and Bolukbasi^18^ applied aluminizing at 700 °C for three hours to WAAM-fabricated Inconel 625 and ER307 stainless steel, demonstrating notable reductions in residual stress, moderate hardness gains, and improved oxidation resistance under elevated-temperature conditions.

The novelty of this study lies in the systematic evaluation of aluminizing as a post-processing route for Wire Arc Additive Manufacturing (WAAM)–fabricated Inconel 625 and ER307 stainless-steel structures, with an emphasis on the relationship between microstructural evolution and corrosion behavior. Unlike previous works that primarily focus on coating development, this study provides an integrated assessment of phase formation, layer-to-layer microstructural gradients, and corrosion performance in chloride-containing environments. Therefore, the present study investigates the effect of an aluminizing treatment performed at 700 °C for 4 h on WAAM-produced ER307 stainless steel and Inconel 625. The study primarily aims to assess how this surface modification influences the corrosion resistance of the alloys in a 3.5% sodium chloride (NaCl) solution.

## Experimental methods

### Fabrication of WAAM components

In this study, wall structures composed of 307 stainless steel and Inconel 625 were fabricated via the Arc-DED process. The fabrication utilised ER307 solid wire conforming to AWS A5.9 and ERNiCrMo-3 (Inconel 625) solid wire conforming to AWS A5.14, both with a diameter of 1.2 mm, as feedstock materials. The chemical compositions of the wires, determined by X-ray fluorescence (XRF) analysis, are presented in Table 1. The mechanical properties of the all-weld metal, as specified in the supplier’s certificates, are summarized in Table 2.

The deposition of both ER307 stainless steel and Inconel 625 wall structures was performed using a Gas Metal Arc Welding (GMAW) power source integrated with the robotic Wire Arc Additive Manufacturing setup. As described in previous research^16–19,23^, the fabrication process was carried out using the GeKa-Tec WB 500 L equipment, which features a water-cooled welding torch mounted on a six-axis OTC Daihen D-V8L robotic system. The WAAM process parameters used during sample production are listed in Table 2. The process parameters were selected based on the deposition results obtained through trial-and-error optimization in our previous study^16,19^. In summary, the deposition directions of successive layers were alternated, with the initial layer deposited in a clockwise orientation. It is well-documented in the literature that the interlayer cooling time critically influences the thermal history and temperature distribution of thin-walled structures manufactured via WAAM. Accordingly, a dwell time of 120 s was introduced following the deposition of each layer to facilitate adequate heat dissipation into the surrounding environment. During the fabrication of the ER307 wall (30 layers), the process parameters were set to an arc voltage of 14.7 V, an arc current of 120 A, and a travel speed of 50 cm/min. For the Inconel 625 wall (30 layers), the parameters included an arc current of 150 A, an arc voltage of 15.8 V, and a travel speed of 50 cm/min. In both cases, a shielding gas mixture consisting of 97.5% argon and 2.5% carbon dioxide was employed at a flow rate of 15 L/min.

**Table 1:** The chemical compositions of the wires utilized in the WAAM process.

Sample	Ni	Cr	Mo	Nb	Mn	Si	Cu	C	Fe
Inconel 625	64.86	21.15	8.67	1.15	–				Balance
ER307	8.95	17.05	0.03		6.18	0.56	0.31	0.05	Balance

**Table 2:** The mechanical properties of the wires utilized in the WAAM process.

Sample	Yield	Ultimate tensile	Elongation	Charpy impact toughness (J at 20 °C)
ER307	432	646	35	105
Inconel 625	540	800	38	–

**Fig. 1:**
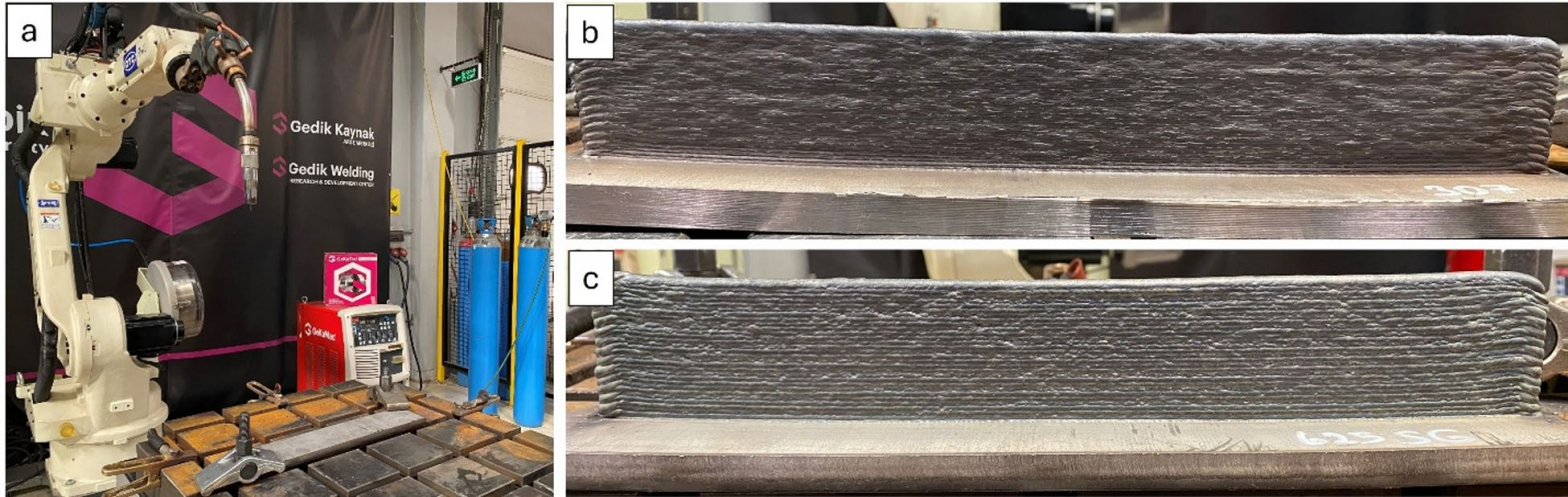
WAAM setup (**a**), (**b**) a general view of the ER307 wall, and (**c**) an overall view of the Inconel 625 wall.

Metallic components measuring 350 × 75 × 12 mm were produced from both materials by WAAM. Figure 1 presents the WAAM setup (a), a general view of the ER307 wall, and an overall view of the Inconel 625 wall.

Due to the high as-built surface roughness, which was approximately 80 ± 5 μm and unsuitable for coating applications, the parts were sectioned into 50 × 50 × 5 mm specimens. These were then subjected to precision milling and surface grinding to reduce the surface roughness below R_a_ < 5 μm. Based on our previous work^18,19^, the powder mixture for aluminizing was prepared using 25% pure aluminium powder as the Al source, 5% ammonium chloride (NH₄Cl) as a halide activator, and 70% aluminium oxide (Al_2_O_3_). The aluminizing treatment was performed by placing the specimens in tightly sealed cylindrical crucibles and heating them in a furnace at 700 °C for 4 h. This treatment was designed to achieve a thicker coating layer compared to earlier studies^18,19^.

**Table 2:** Process parameters employed in the WA-DED production.

Sample	Current (A)	Volt (V)	Speed (mm/sn)	Shielding gas	Gas flow rate (L/min)
Inconel 625	150	15.8	500	97.5%Ar + 2.5% CO_2_	15
ER307	120	14.7	500	97.5%Ar + 2.5% CO_2_	15

Following the aluminizing process, the aluminized samples with initial dimensions of 50 × 50 × 5 mm were sectioned into 20 × 20 × 5 mm pieces for SEM, X-ray diffraction, and corrosion testing. The specimens underwent vertical sectioning to facilitate subsequent analysis. Conventional mechanical grinding and polishing procedures were meticulously applied to attain a clear and well-defined cross-sectional appearance. Notably, the etching process was deemed unnecessary, as the aluminide coatings displayed remarkable visibility under an optical microscope. Subsequent in-depth investigations of the microstructures within the samples were conducted utilizing a scanning electron microscope (SEM). In conjunction with SEM, Energy Dispersive Spectroscopy (EDS) analyses were performed to obtain a comprehensive understanding of the morphology, microstructure, and chemical composition of the specimens. These detailed examinations were carried out utilizing a Circular Backscatter (CBS) detector, employing an accelerating voltage of 10 kV and a spot size of 12 mm to enhance imaging precision.

To verify the existing phases present in the samples, X-ray diffraction (XRD) analyses were performed using a computer-controlled Rigaku SmartLab diffractometer. The analysis employed Cu K_α_ radiation (λ = 1.54 Å), with a 2θ range set from 30° to 90°. The diffractometer was configured with a step size of 0.02° and a scan speed of 2.0° per minute to ensure high-resolution data collection. The XRD results obtained were meticulously cross-referenced for phase identification using PDXL software, facilitating accurate phase analysis. Microhardness assessments were executed on the cross-sectional areas of both the as-built and aluminized Wire Arc Additive Manufacturing (WAAM) specimens. These tests were conducted leveraging a Future-Tech micro-hardness testing device, which was equipped with a Vickers pyramid indenter. The microhardness measurements adhered to the ASTM standards (document number E384), employing a loading force of 100 g with a dwell time of 15 s to ensure precision in hardness evaluation. Furthermore, surface roughness (Ra) was quantitatively assessed using a Wave System Hommelwerke T8000 2D profilometer. The evaluation was conducted with a scan length of 4.8 mm while maintaining a scanning speed of 2 mm/s, ensuring accurate and reliable surface characterization.

For electrochemical testing, corrosion specimens were extracted from the central region of the WAAM-fabricated blocks; each sample was cut from the middle of the 50 × 50 × 5 mm sections to obtain pieces with final dimensions of 20 × 20 × 5 mm. The corrosion characteristics of both as-deposited and aluminized specimens were evaluated through electrochemical corrosion testing conducted in a 3.5 wt% by sodium chloride (NaCl) solution, following the ASTM G106 standard. This assessment primarily employed two techniques: open circuit potential (OCP) measurements and Tafel extrapolation analysis. OCP values were meticulously recorded over 3600 s to ascertain the corrosion potential of each specimen. Subsequently, Tafel extrapolation was executed within a potential window of − 250 mV to + 250 mV relative to the OCP, utilizing a scan rate of 0.166 mV/s. This methodological approach facilitated the determination of the corrosion current density and the corresponding corrosion rates, as delineated in prior research^24^. The exposed surface area during the corrosion assessments was precisely measured at 0.786 cm². Within the experimental setup, a silver/silver chloride (Ag/AgCl) electrode served as the reference electrode, with the specimen functioning as the working electrode (cathode) and a platinum wire acting as the counter electrode (anode). Corrosion tests were performed in triplicate, and for the OCP and Tafel curves, the graph of the sample with the data closest to the mean value for each sample is presented. Following the electrochemical evaluations, the corroded surfaces were subjected to a thorough examination by SEM and EDS to elucidate the specific corrosion mechanisms that occurred. Rp was calculated from the anodic and cathodic Tafel slopes (βa and βc) and the corrosion current density (Icorr) obtained from Tafel extrapolation, using the Stern–Geary relationship^25^:

1 $${{\text{R}}_{\text{p}}}=\frac{{{\upbeta _{\text{a}}} \times \upbeta }}{{2.303 \times {\text{Icorr}}({\upbeta _{\text{a}}}+\upbeta )}}$$

and the corrosion rate (CR) was calculated using the corrosion current density (Icorr) extracted from Tafel extrapolation according to Faraday’s law^26^:

2 $${\text{CR}}=\frac{{{\text{K}} \times {{\text{I}}_{{\text{Coor}}}} \times {\text{EW}}}}{{\text{P}}}$$

Where CR : Corrosion rate (mm/year), K : Constant (3.27 × 10⁻³ mm·g/µA·cm·year), I₍corr₎ : Corrosion current density (A/cm²), EW : Equivalent weight (g/equivalent), ρ : Density (g/cm³).

## Results and discussion

### Microstructural aspects

**Fig. 2:**
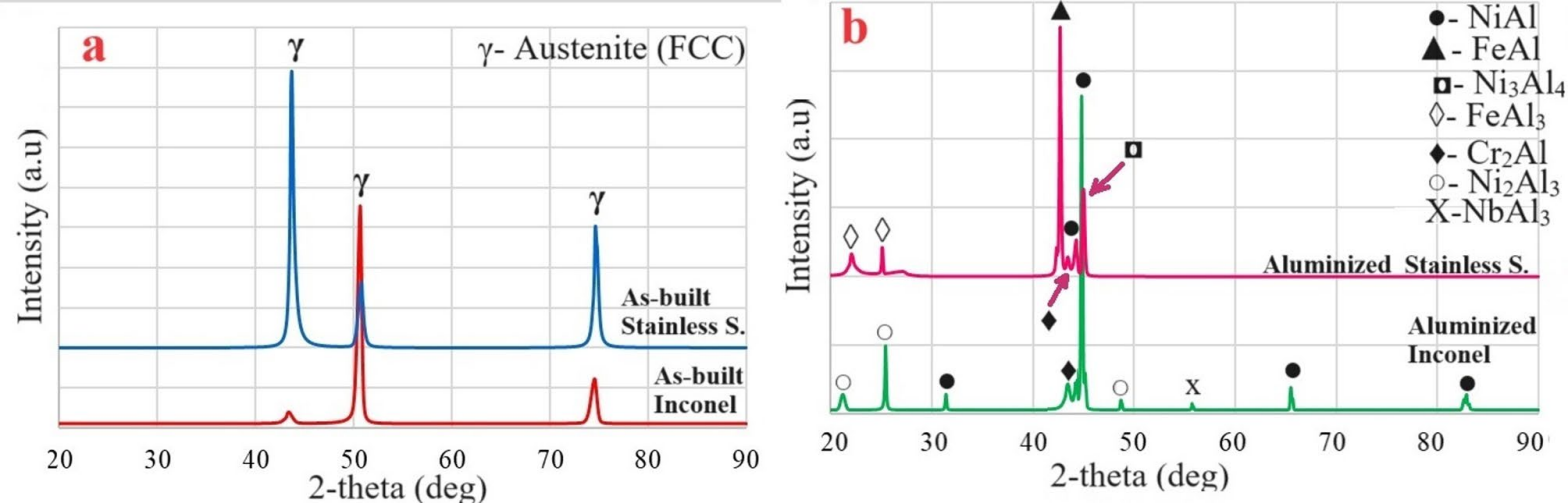
XRD patterns of WAAM samples (**a**) as-built and (**b**) aluminized.

X-ray diffraction (XRD) analyses (Fig. 2) revealed that γ face-centered cubic (FCC) phase peaks appeared at 43.66°, 50.65°, and 74.52° in both as-built Inconel 625 and stainless-steel specimens, with the dominant peak differing between alloys: Inconel exhibited its primary peak at the (200) plane (50.65°), whereas stainless steel showed its strongest diffraction intensity at the (110) plane (43.66°). Following aluminizing, WAAM-fabricated Inconel 625 exhibited NiAl as the predominant coating phase at 44.82°, accompanied by minor phases such as Ni₂Al₃, Cr₂Al, and MoAl₅, whereas aluminized WAAM stainless steel displayed FeAl as the dominant phase at 42.68°, along with smaller fractions of NiAl, Cr₂Al, and FeAl₃; these observations align well with previously reported WAAM-based our studies^18,19^. Although overlapping diffraction peaks hindered precise quantitative phase estimation, relative intensity interpretation suggested that NiAl and Ni₂Al₃ constitute the principal aluminide phases, while Cr₂Al- and Fe-rich phases were present in smaller amounts. The coexistence of multiple aluminide phases may introduce lattice mismatch at phase boundaries, potentially leading to microstructural stress concentrations. Nevertheless, the absence of continuous cracking indicated that the aluminide layer remained mechanically stable. SEM observations revealed localized porosity within the coating, likely arising from aluminum vaporization and diffusion pathways during thermochemical exposure. While such porosity may initially facilitate electrolyte ingress and influence corrosion response, the subsequent formation of a stable alumina-based passive film is expected to mitigate long-term degradation.

**Fig. 3:**
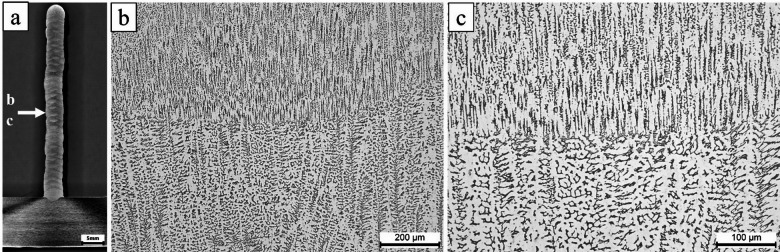
The optical microstructure of the central region of the ER307 stainless steel sample fabricated by WA-DED at different magnifications: (**a**) macro view, (**b**) 100× and (**c**) 200×.

**Fig. 4:**
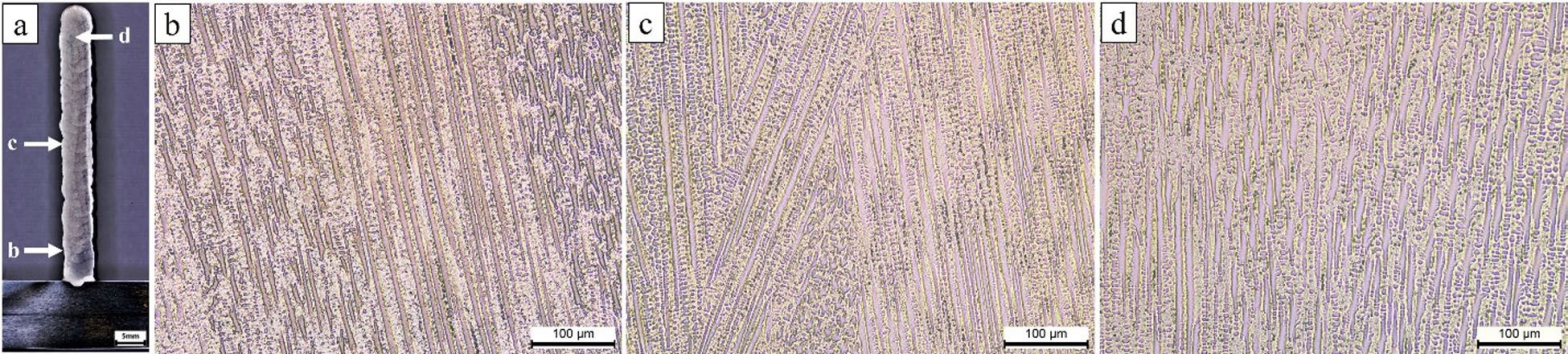
Microstructures of the as-deposited Inconel 625: (**a**) macro view (**b**) bottom, (**c**) middle and (**d**) the top areas of the building direction.

Figures 3 and 4 illustrate the microstructures of the as-deposited ER307 stainless steel and Inconel 625 specimens, respectively.

As observed in Fig. 3a and b, the microstructure of the as-built sample displays a typical dendritic morphology, a characteristic frequently encountered in stainless steels joined through arc or laser beam welding techniques^27–32^. This dendritic structure appears uniformly distributed along the build direction, extending from the bottom to the top of the sample. No macroscopic defects were observed along the build direction during visual inspection, and the selected regions are representative of the bottom, middle, and top portions of the deposited walls, indicating adequate metallurgical bonding throughout the structure. In the micrographs, the light-toned regions correspond to the austenitic phase, while the darker zones indicate the presence of δ-ferrite.

At the upper part of the deposited layer, a refined columnar grain structure dominates, where needle-like δ-ferrites are oriented perpendicularly within an austenitic (γ) matrix. This microstructural configuration is attributed to the high cooling rates prevalent in the remelted areas, which initiate solidification within the bulk volume^33^. Conversely, in the lower sections of the interface, the presence of δ-ferrite is notably reduced, with globular, skeletal, and lath-like formations appearing due to relatively slower cooling rates in these regions compared to the upper interface.

Figure 4 depicts the microstructure of the as-deposited Inconel 625 specimen. Unlike the additively manufactured stainless steel specimen, the microstructure of the Inconel 625 sample is not uniform along the build direction (from bottom to top). The lower section primarily exhibits a fine cellular pattern, a result of the strong cooling effect from the substrate. In contrast, the middle and upper sections show a characteristic columnar dendritic grain structure, with elongated and cellular patterns. As suggested by Yangfan et al.^34^, the microstructural variations observed in different regions of the as-built specimens are attributed to varying cooling rates and heat dissipation during the layer deposition process. Despite these variations, the overall dendritic grain structure remains consistent throughout the entire as-deposited specimen.

Considering Figs. 3 and 4, there are differences in microstructural morphology resulting from layer-by-layer deposition along the build direction during the WAAM process. The bottom region exhibited finer cellular dendrites due to higher thermal dissipation into the substrate, whereas the intermediate region showed a transition toward coarser columnar dendrites formed under reduced thermal gradients. Additionally, partially remelted zones were identified near interlayer boundaries, reflecting reheating during subsequent deposition passes. Localized segregation of Nb- and Mo-rich constituents at interdendritic regions was observed, consistent with repeated thermal cycling. In ER307 stainless steel, the presence of chromium-depleted zones adjacent to δ-ferrite was noted, which is known to increase susceptibility to corrosion in chloride-containing environments^35^. These findings corroborate the strong thermal influence inherent to WAAM fabrication and indicate nonuniform microstructural development along the build height.

### Analysis of aluminide layers

**Fig. 5:**
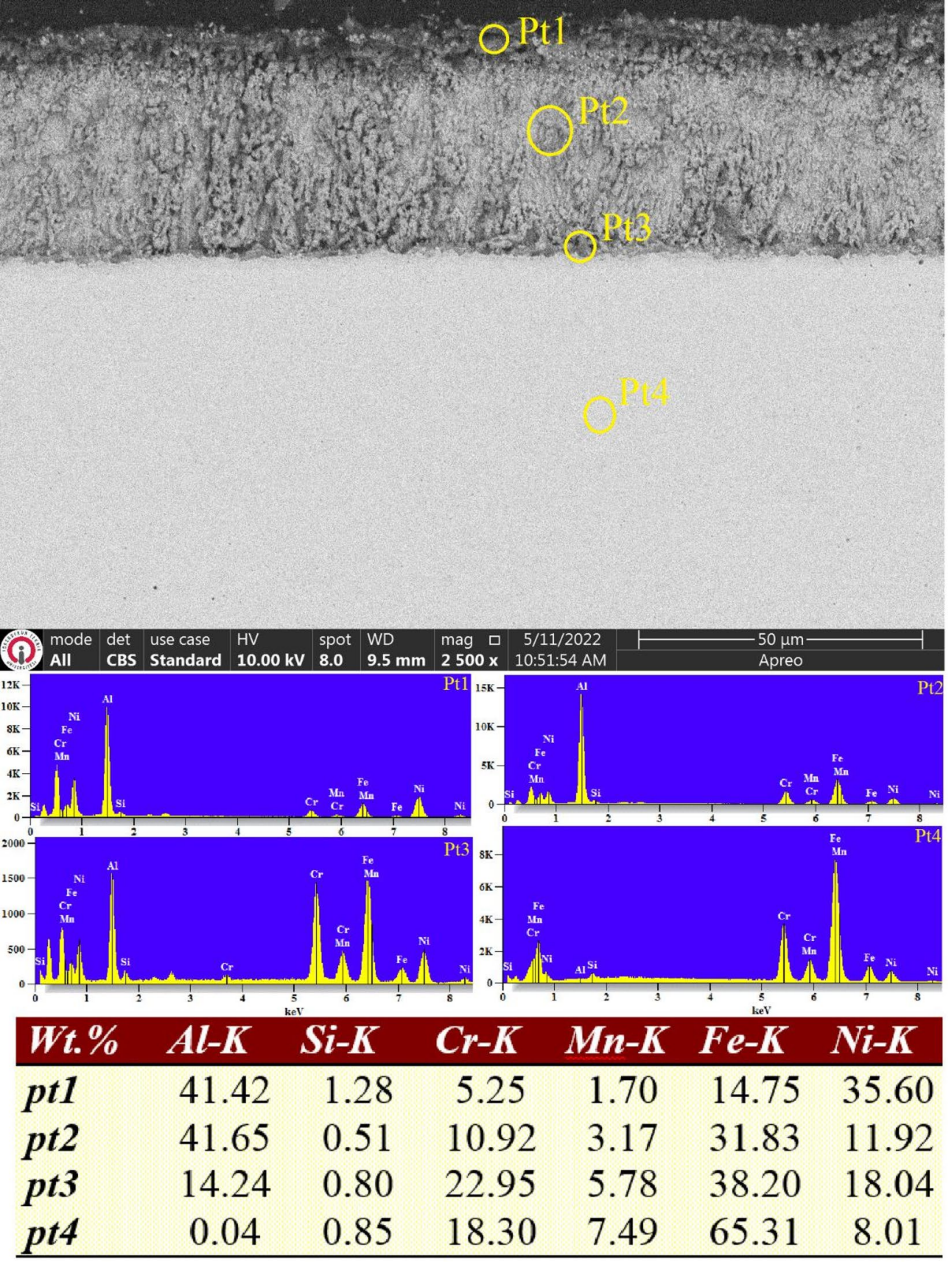
SEM micrograph and EDS point analysis conducted in the cross-section of aluminized ER307 stainless.

**Fig. 6:**
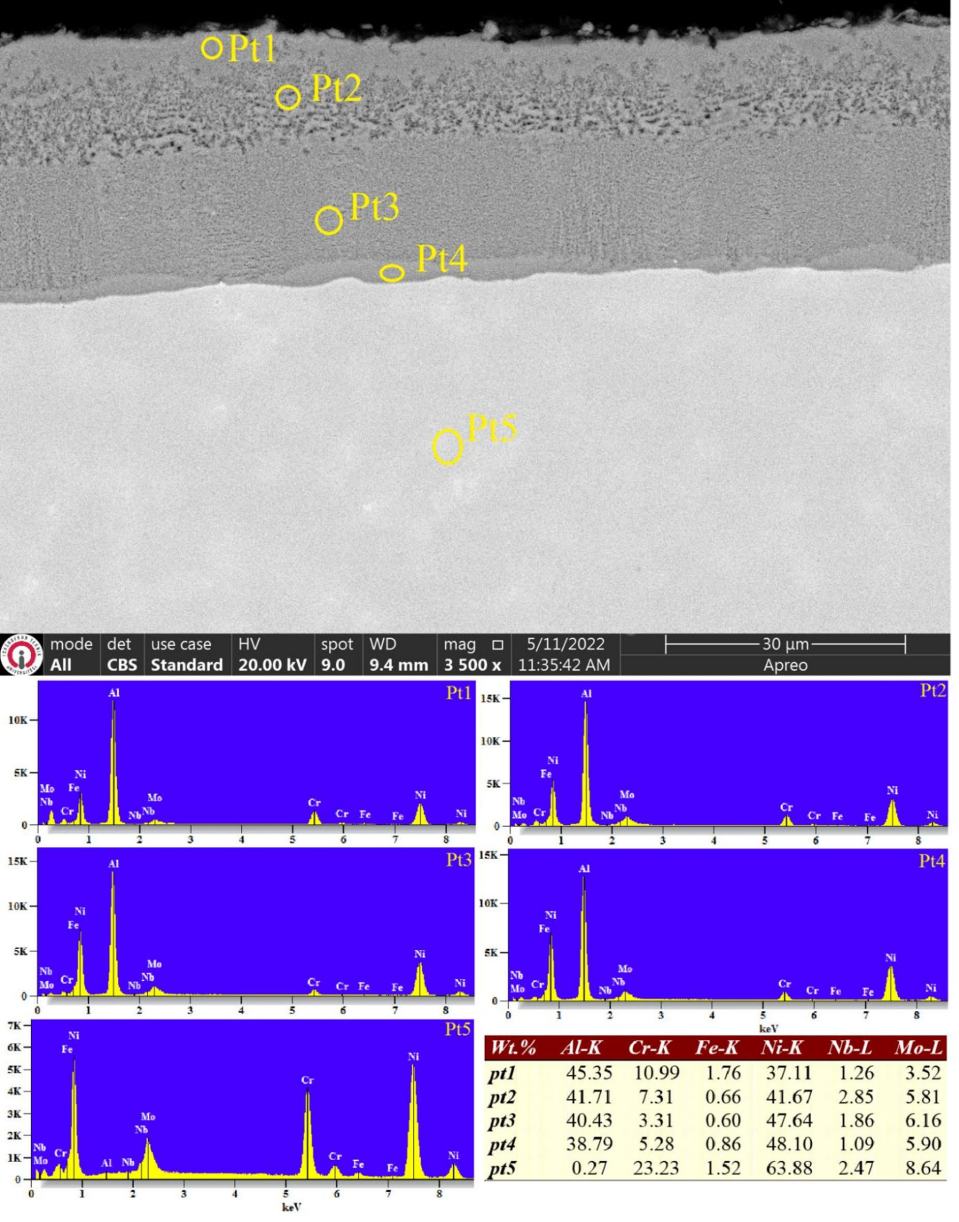
SEM image and EDS point results of aluminized WAAM Inconel 625 alloy.

The cross-sectional SEM images and corresponding EDS point analyses of the samples that underwent aluminizing at 700 °C for four hours are presented in Figs. 5 and 6. As shown in Fig. 5, the WAAM-fabricated ER307 stainless steel samples coated via aluminizing exhibit a single-layer aluminide coating, approximately 50 ± 2.6 μm in thickness, with a surface that displays a noticeably rough morphology. In comparison with the coating structure reported in the earlier work by^19^, this layer demonstrates a higher degree of porosity. This difference is likely attributed to the use of an aluminizing powder mixture containing a lower concentration of aluminum, as explained further below. According to the EDS analysis, the aluminium content at Pt1 and Pt2 points within the coating layer was found to be around 41%, whereas the Pt3 point closer to the substrate exhibited 14% Al. These values suggest that the Pt3 area corresponds to the indistinct transition zone observed in the SEM image, with an estimated thickness of approximately 2–3 μm. As widely noted in the literature, this transition or diffusion zone plays a crucial role in thermochemical coatings by ensuring strong adhesion between the coating and the substrate^18,19^.

**Table 3:** Microhardness, surface roughness, and coating thickness values of the specimens.

Sample	Hardness (HV_0.1_)	Ra (µm)	Rz (µm)	Coating thickness (µm)
Asbuilt ER307	305 ± 15	4.77	< 20	–
Asbuilt ER625	225 ± 15	4.95	< 20	–
Aluminized ER307	1301 ± 27	2.57	< 5	50 ± 2.6
Aluminized ER625	1310 ± 32	2.38	< 5	41 ± 2.2

The aluminizing treatment not only altered the surface chemical composition but also led to a substantial enhancement in microhardness, increasing the value of the WAAM-fabricated stainless steel from 305 ± 15 HV0.1 to 1301 ± 27 HV0.1. Moreover, the average surface roughness (R_a_), initially reduced to 4.77 μm through grinding before coating, further declined to 2.57 μm following aluminizing. The R_z_ value, which was less than 20 μm before the treatment, decreased to below 5 μm after coating (Table 3). This indicates that aluminium particles effectively filled the grinding-induced surface pores, resulting in a significantly smoother finish.

The cross-sectional SEM image of the aluminized Inconel 625 Ni-based alloy, as illustrated in Fig. 6, reveals the formation of a two-layered coating. This dual structure comprises an outer aluminide coating layer with a thickness of approximately 41 ± 2.2 μm, along with a transition zone measuring around 4–5 μm between the coating and the underlying substrate. This configuration contrasts with the single-layer coating observed on the aluminized ER307 stainless steel sample. When compared to the coating thickness reported in earlier studies^18,19^, it becomes apparent that the layer thickness has increased, likely as a result of the extended aluminizing duration. However, this prolonged treatment also appears to have introduced some degree of porosity on the coating surface.

The emergence of porosity may be attributed to the composition of the powder mixture used for aluminizing, which contained 25% aluminum instead of 30%. Additionally, the reduction in vapour concentration due to the lengthened aluminizing time could have led to aluminum diffusion from the coating into the substrate, further contributing to the porosity. EDS analysis confirmed that the aluminum concentration at Pt1 - positioned at the coating surface - was 45% by weight, while this value gradually declined to 38.79% toward the interior regions. Conversely, the nickel content demonstrated an increasing trend from the coating surface inward. This compositional gradient supports the interpretation that aluminum diffused from the surface into the substrate, particularly as aluminum vapour concentration diminished in the reaction environment over time.

Unlike the previous study^18^, where the aluminum distribution across the coating layer was inconsistent, the current sample demonstrates a more gradual decrease in aluminum content. Despite this diffusion effect, the aluminized Inconel 625 surface still exhibited enhanced hardness. Specifically, surface hardness increased to 1310 ± 32 HV0.1, attributed to the presence of nickel aluminide phases, while the hardness of the asbuilt Inconel 625 was 225 ± 10 HV0.1. Concerning surface roughness, measurements indicated that the average Ra value, initially lowered to 4.95 μm through grinding before aluminizing, further decreased to 2.38 μm after the coating process. Similarly, the R_z_ value was reduced from 20 μm to less than 5 μm, mirroring the results observed for the WAAM-fabricated ER307 stainless steel sample (Table 3). The significant decrease in Ra and Rz values ​​of both WAAM ER307 and WAAM Inconel 625 after aluminizing is thought to be a result of partial recrystallization resulting from thermally activated surface diffusion at 700 °C. This suggests a complementary mechanism for the observed decrease in Ra and Rz.

### Results of electrochemical corrosion tests

**Fig. 7:**
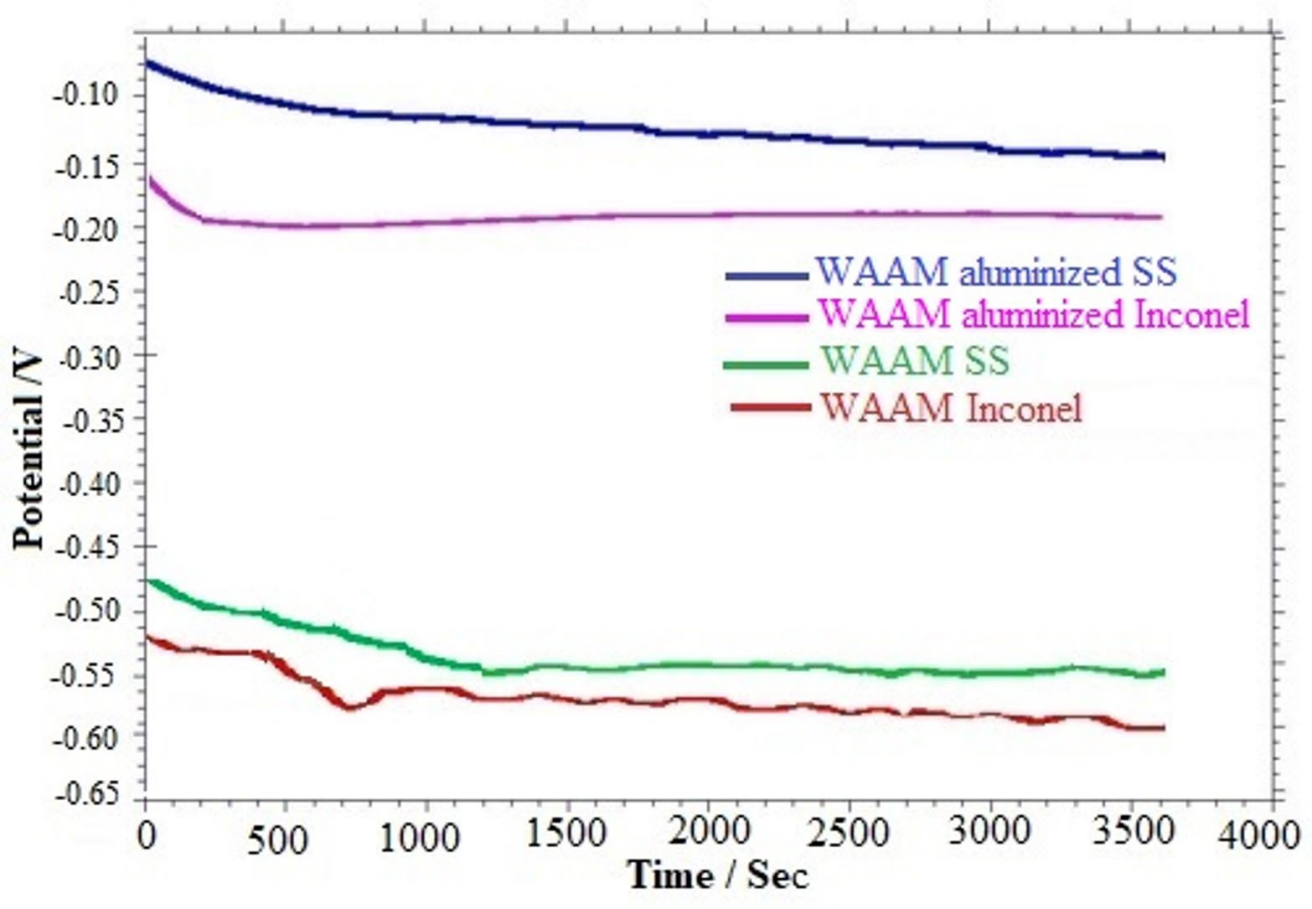
OCP results of WAAM-samples in 3.5% NaCl solution.

Electrochemical corrosion tests were performed on both as-built and aluminized specimens fabricated via the WAAM method by immersing them in a 3.5% sodium chloride (NaCl) solution. The primary objective of these tests was to assess the corrosion resistance of the samples by monitoring the open circuit potential (OCP) and interpreting the potential profiles obtained through Tafel extrapolation, which reflects the anodic and cathodic electrochemical reactions occurring in the test environment. The OCP curves corresponding to the as-built and aluminized specimens immersed in the 3.5% NaCl solution are presented in Fig. 7.

A detailed evaluation of Fig. 7 reveals that the open circuit potential (OCP) values for the as-deposited ER307 stainless steel and Inconel 625 samples fall within the range of − 0.48 to − 0.60 V. In contrast, the aluminized samples display significantly more noble behavior, with OCP values ranging between − 0.10 and − 0.20 V. In these coated specimens, the OCP exhibited a gradual decline during the initial 500 s of immersion, ultimately reaching a stable state. However, while the as-built ER307 stainless steel sample achieved a quasi-steady potential around 1500 s, the as-built Inconel 625 alloy failed to stabilize even after prolonged exposure, indicating its comparatively inferior electrochemical stability under the tested conditions.

This observation suggests a higher degree of elemental segregation in the WAAM-fabricated Inconel 625 alloy relative to the ER307 sample, likely stemming from its richer alloying element content and the elevated thermal input inherent to the WAAM process. Such segregation phenomena have been widely reported in the literature and are known to compromise corrosion resistance^36,37^ The susceptibility of Inconel 625 to corrosion under open-circuit conditions can therefore be attributed to these microstructural inhomogeneities. It has also been emphasized in previous studies that post-deposition heat treatments can alleviate these adverse effects by promoting elemental homogenization and microstructural refinement^23,38,39^. Furthermore, the OCP behavior of the aluminized specimens underscores the efficacy of the aluminide layer in mitigating the corrosive attack. Acting as a chemically stable and physically barrier, this coating impedes the penetration of chloride ions from the NaCl solution, thereby reducing direct interaction with the underlying substrate and enhancing corrosion resistance.

**Fig. 8:**
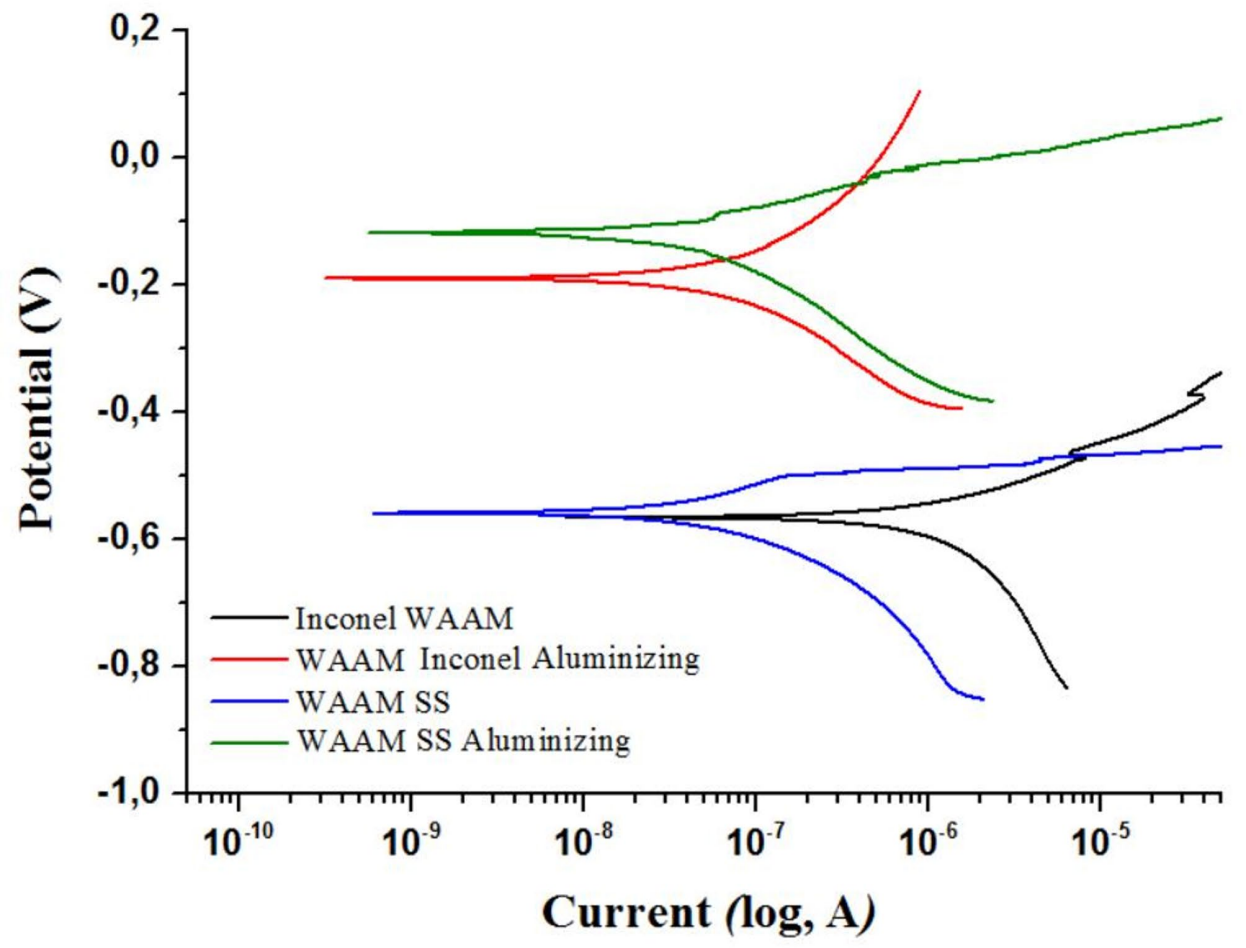
Tafel extrapolation results of WAAM-samples in 3.5% NaCl solution.

An inspection of the Tafel polarization curves presented in Fig. 8 reveals a notable shift in electrochemical parameters following aluminizing. The coated samples demonstrate a displacement of corrosion potential (E_corr_) towards more positive values, accompanied by a reduction in corrosion current density (I_corr_), as evidenced by their logarithmic current values trending towards more negative regions. These shifts indicate a broadening of the passive region and reduced corrosion kinetics. Collectively, these findings affirm that the aluminizing process significantly enhances the corrosion performance of both ER307 stainless steel and Inconel 625 alloys produced via Arc-DED.

The observed improvements in corrosion resistance can be attributed to the expansion of the passive region and the shift in corrosion potential toward more noble (positive) values in the aluminide-coated specimens^40^. Specifically, these coatings not only enhanced electrochemical stability but also resulted in a noticeable reduction in corrosion kinetics. This is evidenced by the leftward shift of the logarithmic polarization curves along the x-axis, indicating lower corrosion current densities. For instance, the aluminized ER307 stainless steel sample exhibited both a more positive corrosion potential and a significantly lower I_corr_ value when compared to its uncoated counterpart. Together, these findings demonstrate that among the evaluated samples, the aluminized ER307 stainless steel specimen possesses the most favourable corrosion resistance characteristics.

For a more comprehensive understanding of the corrosion behavior, quantitative electrochemical parameters - namely the corrosion potential (E_corr_), corrosion current density (I_corr_), corrosion rate (expressed in mils per year, mpy), and polarization resistance (R_p_)—obtained from the OCP and Tafel plots are presented in Table 4.

**Table 4:** Summary of corrosion data obtained from OCP and Tafel curves.

Sample	E_corr_ (mV)	I_corr_ (nA)	Corrosion rate (mpy)	*R*_*p*_ (kΩ)
As-built Inconel 625	− 564 ± 21	1680 ± 64	9.79 ± 0.72	22.53 ± 1.72
As-built ER307 SS	− 557 ± 14	41 ± 1.7	0.24 ± 0.15	172.0 ± 8.5
Aluminized Inconel 625	− 189 ± 12	120 ± 8	0.70 ± 0.08	431.5 ± 25.40
Aluminized ER307	− 118 ± 6	32 ± 2	0.19 ± 0.02	398.7 ± 15.72

An evaluation of Table 4 shows that the as-deposited Inconel 625 exhibited the lowest corrosion potential (Ecorr) and polarization resistance (Rp), resulting in the highest corrosion rate of 9.79 mpy, which is attributed to elemental segregation inherent to the WAAM process. Following aluminizing, the corrosion rate of Inconel 625 decreased markedly to 0.70 mpy, corresponding to an approximately 14-fold improvement, consistent with the significant increase in Rp from 22.53 ± 1.72 kΩ to 431.5 ± 25.40 kΩ. This improvement was further supported by the reduction in corrosion current density (Icorr) from 1680 ± 64 nA to 120 ± 8 nA, indicating comparable inhibition of electrochemical activity. These trends confirm that aluminizing effectively suppresses anodic dissolution and enhances passive film stability. In comparison, the as-built ER307 stainless steel sample showed an initial corrosion rate of 0.24 mpy, which slightly decreased to 0.19 mpy after aluminizing, indicating that its corrosion performance was already superior in the as-deposited state.

The improved corrosion resistance of the aluminized specimens (Table 4) can be primarily attributed to the formation of a chemically stable aluminide layer that restricts chloride penetration and suppresses localized dissolution, in contrast to the as-built condition where elemental segregation and inter-dendritic inhomogeneity promote anodic activity. In line with the observations of Maurya et al., the suppression of micro-galvanic sites and mitigation of segregation-driven susceptibility around Ni- and Mo-rich regions reduce pit initiation and propagation, thereby enhancing electrochemical stability in chloride-containing environments. This improvement can be directly correlated with the microstructural evolution induced by thermochemical diffusion, wherein the formation of NiAl and Ni₂Al₃ intermetallic phases on WAAM-fabricated Inconel 625, together with FeAl-based aluminides on ER307 stainless steel, promotes the development of a reveal localized surface porosity and limited non-uniformity aluminide layer and chemically stable diffusion zone that minimizes energetically favorable anodic dissolution sites. This barrier effect not only restricts chloride ingress and reduces defect-assisted transport pathways—leading to more noble Ecorr values—but also contributes to lower Icorr values through homogenization of Ni-, Mo-, and Nb-rich segregated regions, which otherwise facilitate micro-galvanic activity, and through the establishment of a continuous Al-enriched passive layer that limits electron-exchange kinetics. Moreover, the enhanced Rp values of the aluminized specimens indicate increased resistance to charge transfer, consistent with the presence of stable aluminide phases and reduced porosity. These outcomes highlight clear distinctions in corrosion performance between as-built and aluminized samples, emphasizing the influence of process-related factors such as heat input, interlayer segregation, and surface defects inherent to the WAAM technique, while underscoring the role of coating compactness, phase composition, and Al concentration gradients in governing corrosion behavior; coatings with higher NiAl/Ni₂Al₃ fractions exhibit superior performance due to their thermodynamic stability and slow dissolution rates in chloride environments.

**Fig. 9:**
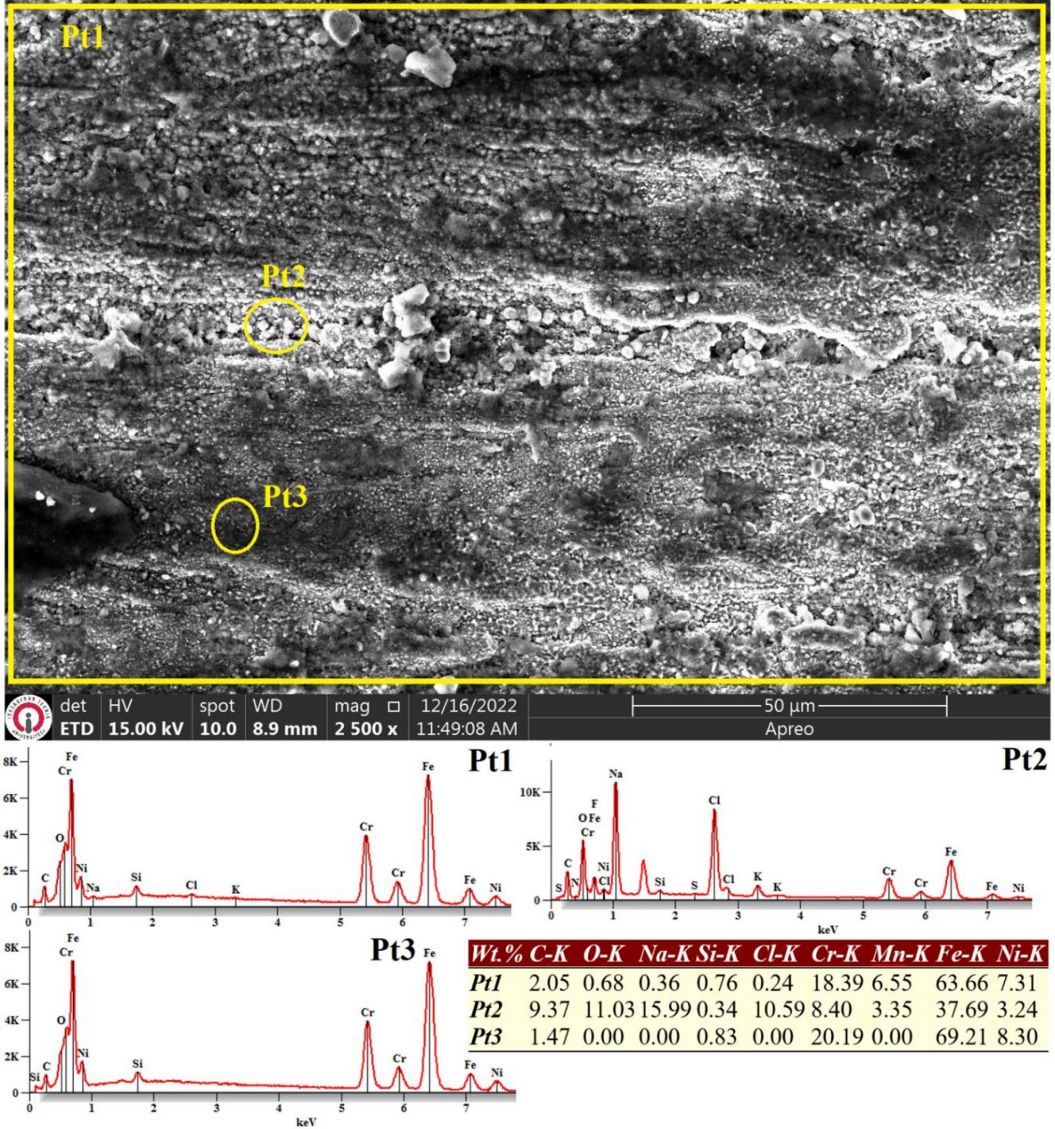
SEM image and EDS analysis results on the surface of as-built WAAM ER307 specimen subjected to electrochemical corrosion test in 3.5% NaCl solution.

As depicted in Fig. 9, the dominant corrosion mechanism observed in the as-built ER307 stainless steel specimen is uniform corrosion across a major portion of the surface. In the regions affected by corrosion (Pt2), oxygen content was measured at approximately 11.03%, whereas it remained significantly lower - ranging from 0 to 0.68% - in darker, less corroded areas (Pt1 and Pt3). The localized presence of sodium and chloride residues in Pt2 further confirms active corrosion in those regions, as these elements were distinctly identified on the surface.

**Fig. 10:**
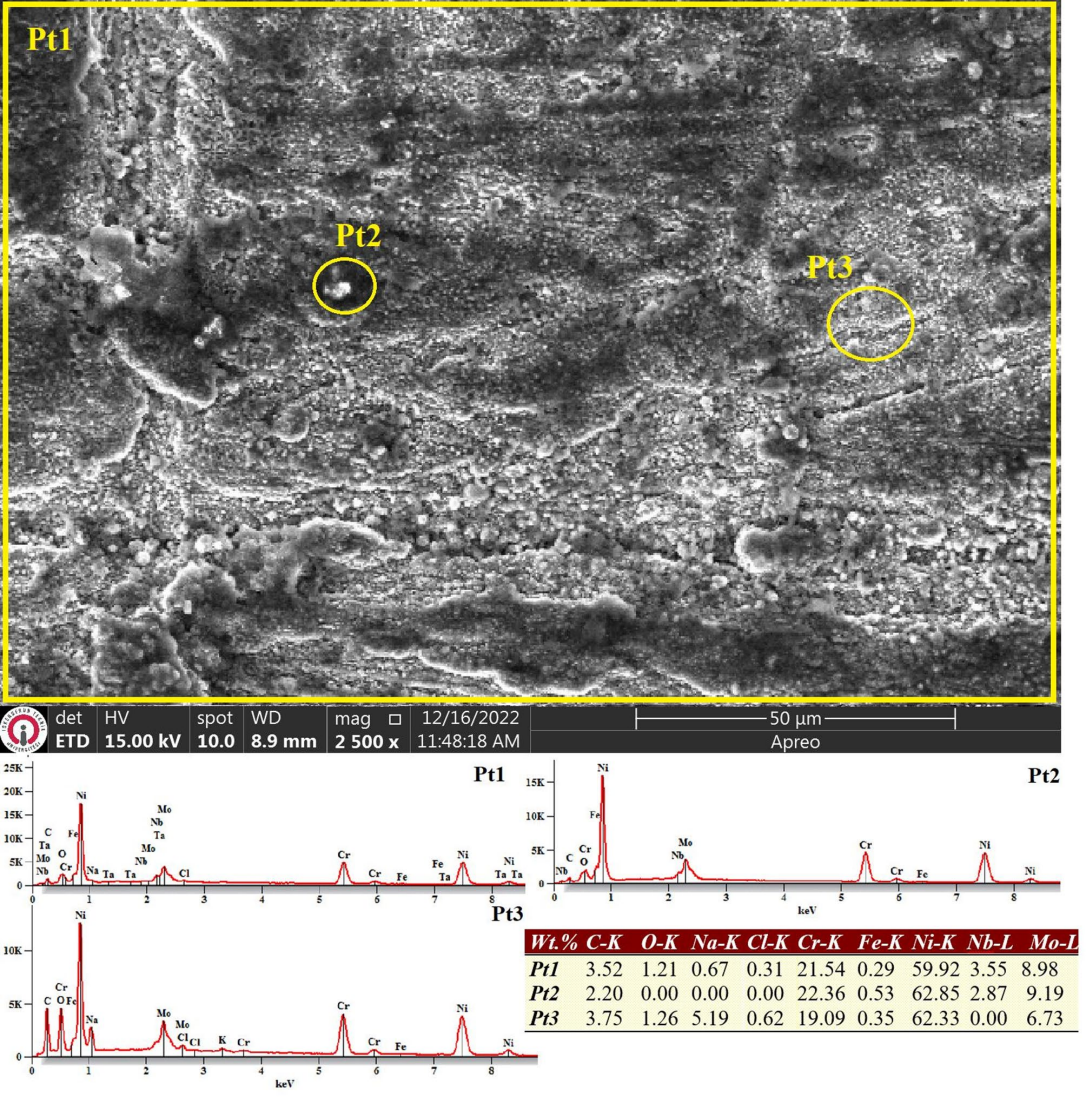
SEM image and EDS analysis conducted on the surface of as-built WAAM ER307 specimen subjected to electrochemical corrosion test in 3.5% NaCl solution.

Analogous to the behavior observed in the as-built WAAM-fabricated ER307 sample, Fig. 10 indicates that homogeneous corrosion remains the dominant mechanism across a substantial portion of the Inconel 625 surface. However, in certain localized regions - particularly beneath Pt3 - microcracks are evident. These microstructural defects may have played a significant role in the reduced corrosion resistance of the sample, as they provide pathways for corrosive agents to infiltrate into subsurface regions, as corroborated by the data presented in Table 4. The corrosion performance of WAAM-produced components is inevitably compromised by the presence of surface porosities formed during fabrication, as well as the structural heterogeneity introduced by elemental segregation and cracking along the build cross-section.

Such defects facilitate the ingress of the corrosive medium into the interior, enabling corrosion reactions to progress along the path created by interconnected pores and cracks^41^. Moreover, comparative EDS analysis reveals a notable disparity in the oxygen content on the corroded surface of Inconel 625 relative to ER307, suggesting differential corrosion dynamics between the two alloys.

**Fig. 11:**
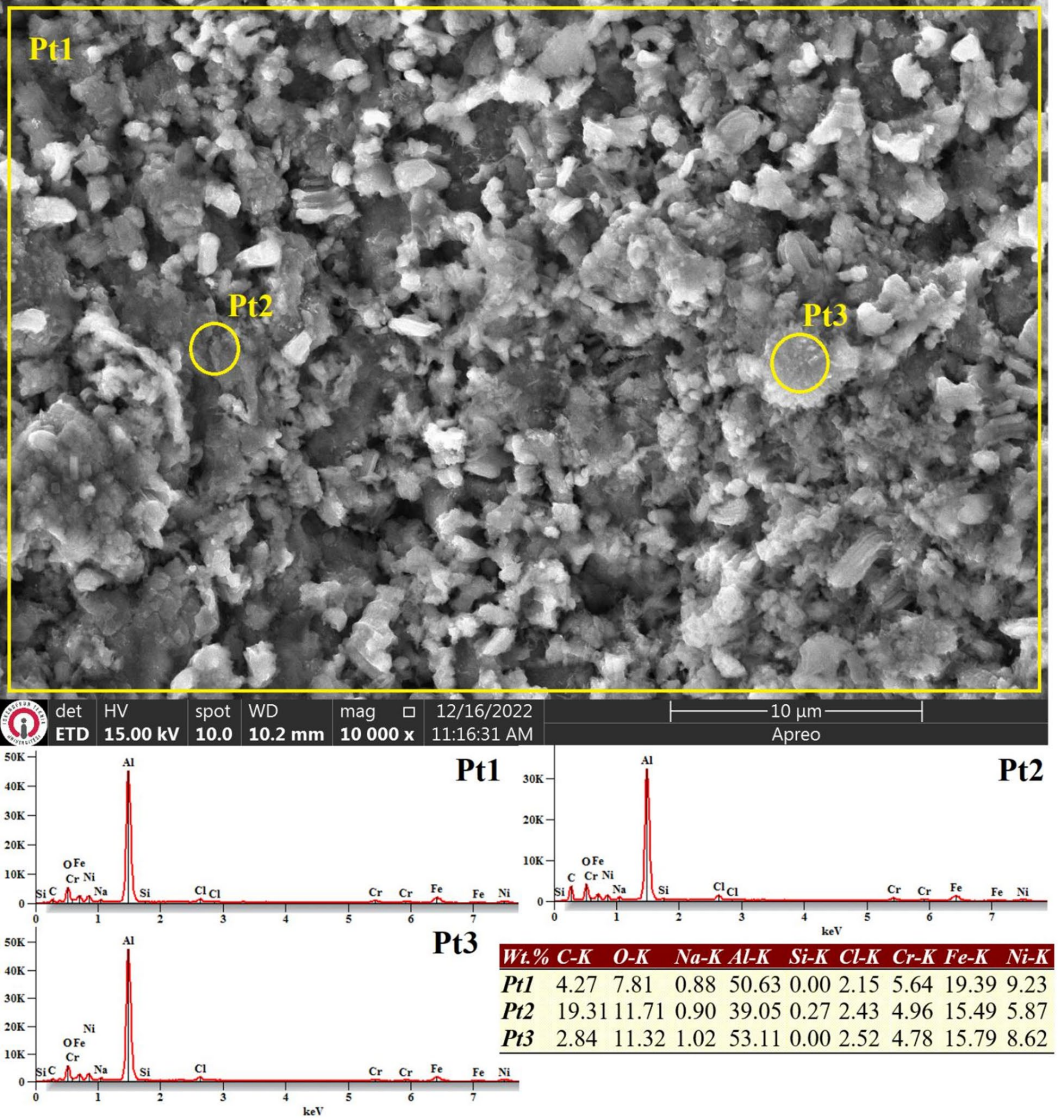
SEM image and EDS analysis conducted on the surface of the aluminized WAAM ER307 sample after undergoing electrochemical corrosion testing in a 3.5% NaCl solution.

Due to insufficient image clarity at a magnification of 2500X in the corroded regions of the aluminized WAAM ER307 specimen, SEM imaging was instead conducted at a higher magnification of 10000X. As illustrated in Fig. 11, the surface displays a structure composed of uniformly distributed, particle-like formations indicative of pitting corrosion. EDS surface analysis revealed aluminum concentrations ranging from 39% to 53% and oxygen levels between 7.81% and 11.71%, confirming that the corrosion was predominantly localized on the aluminide coating layer.

**Fig. 12:**
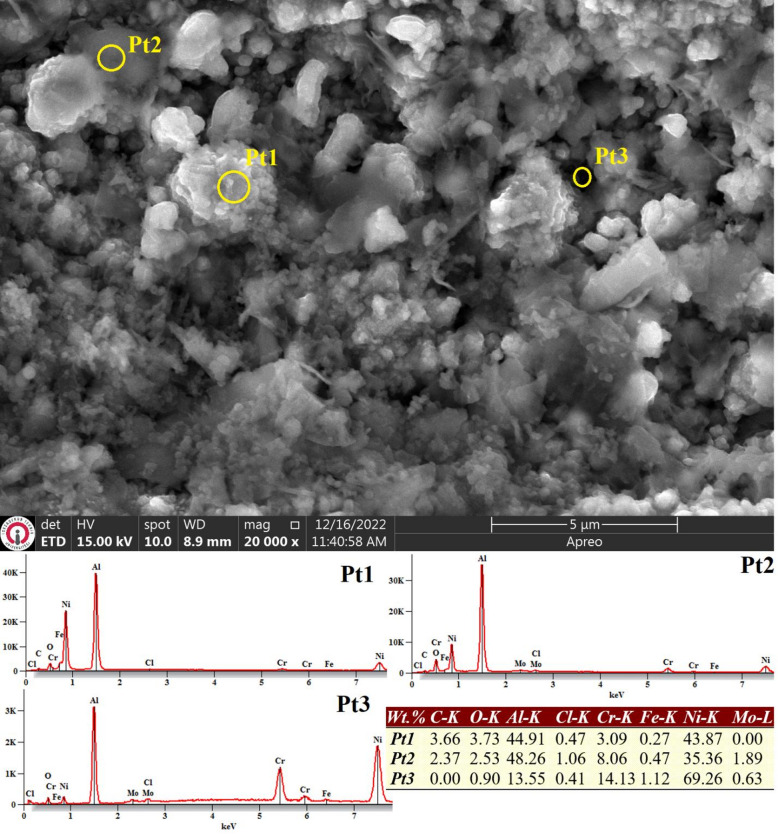
SEM image and EDS analysis conductyed on the surface of the aluminized Inconel 625 sample following exposure to electrochemical corrosion testing in a 3.5% NaCl solution.

Similarly, the post-corrosion surface morphology of the aluminized WAAM Inconel 625 sample closely mirrors that of the aluminized ER307 alloy. Figure 12 illustrates a surface characterized by quasi-homogeneous particulate features interspersed with occasional pores (Pt3). The presence of aluminum (13.55%-48.26%) and oxygen (7.81%-11.71%) in EDS analyses further supports that the corrosion process occurred within the aluminide layer. These findings affirm that the aluminide coating remained adherent and continued to exist on the surface even after exposure to corrosive conditions.

## Conclusions

In this study, ER307 stainless steel and Inconel 625 superalloy wall structures fabricated via Wire Arc Directed Energy Deposition (WA-DED) were subjected to an aluminizing treatment at 700 °C for four hours. The investigation focused on evaluating the effects of the resulting aluminide coatings on the surface properties and corrosion resistance of the WAAM-fabricated components. The principal findings derived from this research can be summarized as follows:

- Microstructural gradients were detected along the build height of the as-built WAAM samples. This transition from fine cellular structures at the bottom to coarser columnar dendrites toward the top is attributable to changes in thermal conditions during the WAAM deposition process.
- The WAAM-fabricated Inconel 625 alloy exhibited lower polarization resistance (R_p_) and higher corrosion current density (I_corr_) values compared to the ER307 stainless steel counterpart. This difference is primarily attributed to the more pronounced elemental segregation present within the microstructure of Inconel 625. While homogeneous corrosion was the dominant degradation mechanism in the as-built ER307 stainless steel, the Inconel 625 alloy displayed both homogeneous and crevice corrosion characteristics.
- Following the aluminizing process, continuous coating layers ranging from 40 to 50 μm in thickness were successfully formed across the sample surfaces. However, localized porosity was observed within the aluminide coatings. This phenomenon is believed to be associated with the relatively low aluminum content used in the powder mixture, combined with the extended duration of the aluminizing treatment.
- The chemically stable aluminide coatings formed on the surfaces served as effective barriers against the ingress of corrosive media. Moreover, the aluminizing process contributed to a refinement of the surface microstructure, particularly for the Inconel 625 samples, by reducing surface porosity and associated defects introduced during WAAM processing.
- The enhanced electrochemical stability of the aluminized samples, as evidenced by their performance in OCP and Tafel polarization analyses compared to untreated as-built counterparts, suggests that these coated materials are more suitable for long-term service in corrosive environments. These results clearly demonstrate that the inherent limitation of poor corrosion resistance in WAAM-fabricated components can be effectively mitigated through aluminizing - a relatively simple and cost-efficient surface treatment method.

## Data Availability

No datasets were generated or analysed during the current study.
